# Supplementary material for: Research on the enhancement path of green technology innovation efficiency under the group perspective
Source: PLoS One. 2024 Aug 15;19(8):e0306936. doi: 10.1371/journal.pone.0306936 (PMC11326581; doi:10.1371/journal.pone.0306936)
Supplement: S1 File — (PDF) [file pone.0306936.s002.pdf]

Author Copy • Author Copy • Author Copy • Author Copy • Author Copy • Author Copy • Author Copy • Author Copy • Author Copy • Author Copy • Author Copy • Author Copy

Pol. J. Environ. Stud. Vol. 33, No. 1 (2024), 1-17
DOI: *10.15244/pjoes/171660* ONLINE PUBLICATION DATE:

Original Research

Analysis of Green Technology Innovation Efficiency Measurement in China's High-Tech Industries

Lei Liu, Li Zhang*, Wei Xu

School of Management, Shenyang University of Technology, Shenyang, Liaoning, China

Received: 26 July 2023
Accepted: 29 August 2023

Abstract

Based on the two-stage innovation value chain model, incorporating environmental regulation policies and three-waste emissions into the research framework, constructing a green technology innovation efficiency evaluation index system from the perspectives of green inputs and outputs, and utilizing the super-efficiency SBM model and the Malmquist-Luenberger index model, assessing the effectiveness of green technological innovation in two aspects, namely, static, and dynamic, respectively. The results show that: the average value of green technical innovation efficiency of China's high-tech industries is 0.5277, with significant differences between provinces, but the overall is constantly improving, showing an upward trend; the green technological innovation efficiency decreases from east to west, and the differences among the three major regions are not only embodied in spatial patterns, but also in the growth rate; during the period of investigation, the inefficient provinces are gradually decreasing, and the level of green technological innovation of China is constantly getting better; China's The average value of ML index of green technical innovation efficiency of high-tech industries in China is greater than 1, and the overall development trend is better; the increase of ML index in the eastern and central regions mainly relies on the positive influence of technological efficiency, while the decrease of ML index in the western part is affected by the recession of technological progress and technological efficiency, and the efficiency of technology research and development stage is generally higher than that of scientific and technical achievements transformation stage, and low efficiency of achievements transformation stage is mainly affected by the inhibiting effect of technological progress – inhibitory effect of technological advancement.

Keywords: green technological innovation, super-efficiency SBM model, Malmquist index, environmental regulation policy

*e-mail: zyali2023@163.com

Author Copy • Author Copy • Author Copy • Author Copy • Author Copy • Author Copy • Author Copy • Author Copy • Author Copy • Author Copy

Introduction

The report of the 20th Party Congress states that technological innovation should be utilized in all aspects of modernization to activate innovation as an economic engine fully. Technological innovation drives a country or region's sustained and healthy economic development, and natural resources and the environment are essential symbols of its sustainable development [1]. The Fifth Plenary Session of the 18th Central Committee paid great attention to environmental issues. It introduced the concept of green development, and the 75th United Nations General Assembly of China put forward the goal of carbon peak and carbon neutrality. To realize the transformation from high-speed development to high-quality, sustainable development, green transformation and upgrading is the way to go. The high-tech industry is a strategic industry for China's economic development and is leading China's high-quality development and industrial transformation; as with traditional technological innovation, technological innovation in the high-tech sector leads to economic growth but also has the potential to bring about problems such as environmental pollution, energy consumption and so on [2]. Therefore, the development of green innovation in high-tech industries should not only consider the problem of innovation ability but also consider how to improve environmental quality and reduce energy consumption while improving efficiency.

In recent years, high-tech industries have been increasing R&D investment, establishing various types of high-tech industrial parks, and expanding the scale of enterprises. At the same time, provinces have encouraged enterprises to transform into green and ecological enterprises to improve green innovation. Among them, the R&D expenditure in the R&D investment stage of high-tech industries has risen from 144.9 billion yuan in 2011 to 464.86 billion yuan in 2020 (see Table 2). However, the economic benefits in the transformation stage of the results have yet to be substantially improved. This indicates that the continuous expansion of enterprise scale does not dramatically improve its innovation efficiency. Instead, it leads to inefficient resource utilization, structural redundancy, and severe environmental pollution, failing to improve the efficiency of green innovation significantly [3]. Instead, it makes resource utilization inefficient, structure redundant, and environmental pollution severe, resulting in green innovation efficiency not significantly improved [4]. In addition, enterprises often need help with obstacles such as high cost and high risk due to government regulations when they carry out green innovation activities, which also indirectly leads to the weak innovation capacity of China's high-tech industry. Therefore, improving efficiency by increasing R&D investment without considering the efficiency of transforming green scientific and technological achievements will inevitably lead to wasting resources [5]. The following are some of the reasons for this. Then, it is worthwhile to explore which parts of the enterprise in the process of carrying out innovation activities could be more problematic, leading to inefficiency, and the reason.

Currently, research on technological innovation is relatively extensive, with many findings exploring influencing factors and measuring and evaluating efficiency. However, there is a complete theoretical framework on innovation efficiency, especially green technology innovation efficiency, which needs to be further improved, and there needs to be more literature on government regulation as an input indicator. Therefore, designing more accurate and suitable evaluation indexes for green technology innovation to assess and measure the innovation level of green technology, analyzing its differences in the development process, digging into the specific reasons for the differences, and supplementing and deepening the evaluation theory of the efficiency of green technology innovation has essential theoretical value for the innovative development of high-tech industries, and also provide transformational ideas for the green development of enterprises.

Literature Review

The concept of green technological innovation originated in Western countries and was first proposed by Braun E and Wield D [6], they believed that if the innovation process can reduce environmental pollution or energy consumption, whether it is technology, product, or process, it can be called green technology innovation. With the continuous consumption of resources by economic development, some Western countries have begun to take the initiative to adjust their technology to the direction of green technology; for example, Ghisetti C., Rennings K. [7] that the reduction of carbon dioxide in the output or the removal of energy consumption in the innovation process belongs to the energy resource efficient innovation, and if the enterprise can reduce the water pollution, air pollution, land pollution and so on in the innovation process belongs to the externality weakening type of innovation. China's research on green technological innovation is developed based on foreign research; He Xiaogang [8] believes that green technological innovation requires enterprises to efficiently utilize limited resources to create revenue, protect the environment, and realize the double leap of resources and the environment. Zhang Feng, Ren Shijia, and Yin Xiucheng [9] believe that green transformation enterprises should focus on the research and development of green technology and develop green products through green technology, so the focus of green technology innovation should be placed on the research and development of green technology to reduce environmental pollution while realizing the improvement of economic benefits. Based on existing literature, this paper argues that green technology innovation should generate knowledge-

Author Copy • Author Copy • Author Copy • Author Copy • Author Copy • Author Copy • Author Copy • Author Copy • Author Copy • Author Copy

based innovations and create as many financial benefits as possible but also uses innovative technology to reduce pollution emissions and realize energy saving and environmental protection. As resources and the environment are constantly emphasized, people pay more attention to the impact of pollution of resources and background on innovation efficiency and gradually shift from pure research on technological innovation efficiency to green technical innovation efficiency. Most scholars focus on green technology innovation efficiency from the perspective of inputs and outputs; Luo Liangwen and Liang Shengrong [10] use the total inputs used in production compared with the total outputs finally obtained to express the green technology innovation efficiency. That is the efficiency of the maximum desired output and the minimum non-desired output obtained by considering the inputs of resource elements such as personnel and capital, examining whether the existing resources are fully utilized and whether the allocation of various factors is used rationally, etc.

Green Technology Innovation Efficiency There are two main methods to measure efficiency: stochastic frontier (SFA) and data envelopment analysis (DEA). Wang Yan, Gong Xinshu, and Li Jinjin [11] use the SFA model to measure the technological innovation efficiency of Xinjiang equipment manufacturing industry; because SFA needs to set up an estimation model, there are constraints in using this method to measure the efficiency, so subsequent researchers have more often used DEA method to measure the efficiency. He Wei [12] evaluated the input and output efficiency of agricultural science and technology parks using the traditional DEA method; Lu Y.H., Shen C.C., Ting C.T. [13] evaluated the R&D efficiency of 194 high-tech enterprises in Taiwan using the traditional DEA method. The conventional DEA only considered the initial and final outputs and did not consider the impact of the links existing in the middle on the efficiency and the problem of slackness between inputs and outputs. To solve this problem, many researchers have proposed network DEA and SBM models [14, 15]. The former divides inputs and outputs into several stages, and relevant intermediate indicators connect each step. At the same time, the latter can consider the slackness neglected by traditional DEA. Luo Wenliang and Liang Shengrong [10] constructed an indicator system using a two-stage DEA model to evaluate the efficiency of green technology innovation of Chinese regional enterprises. Zhu Honghui, Yang Shuqi [16] Using a two-stage DEA model, the innovation capacity of patent-intensive and non-patent-intensive manufacturing industries at each stage was analyzed comparatively. However, the SBM cannot further compare the decision-making units whose efficiency is already 1. Tone [17] continues the research on this model and proposes a super-efficient SBM model that can more accurately compare the efficiency values. Li Hui, Li Wei, and Yao Xilong [18] Measured the total factor carbon emission efficiency of Chinese provinces with a super-efficient SBM model. Xu Yingqi, Cheng Yu, and Wang Jingjing [19] then used the super-efficiency SBM model to measure the carbon emission efficiency of 68 cities in China that have implemented low-carbon pilots. For the defect that the super-efficient SBM model can only estimate the static efficiency at each stage, but cannot measure the dynamic trend, Yan Huafei, Xiao Jing, and Feng Bing [20] think that the Malmquist model can compensate for it. Lai Yifei, Xie Panjia, Ye Liting et al. [21] measured the dynamic efficiency of science and technology innovation in provinces and cities by using the SBM-Malmquist model. Chen Wei, Zhang Changxiao, and Li Chuanyun [22] measured China's high-tech service industry's innovation efficiency by combining the DEA and the Malmquist-Luenberge index. Zhang Likun, Zhang Yaping, Liang Yuan [23] Analyzing Chinese industrial enterprises' green technology innovation efficiency with the Malmquist-Luenberge model.

In evaluating indicators, scholars choose different hands, but basically, they are selected after considering inputs and outputs. Zhao Qiaozhi, Liu Jonas Xuan, Cui, and Rui [24] chose funding and personnel as inputs in the knowledge stage and used the Internet penetration rate as a particular factor input and the sales revenue of new products as the final output. Jaffe A.B., Palmer K.L. [25] chose R&D personnel expenses as the measurement index. At the same time, Sun Yanming and Chen Simiao [26], based on the previous research and considering the resource demand situation, used the total amount of industrial water consumption, the total amount of industrial electricity consumption, and so on to indicate the inputs of resources. With the increasing ease of obtaining patent data, scholars began to use the number of patents to measure innovation performance, Scholars such as Ley M., Stucki T., Woerter M. [27], and Popp D. [28] used the number of patent applications to identify the innovation quality of innovation subjects. Li Dandan [29] believes patents can effectively respond to the transformation of innovation results, so the number of patent applications is an output indicator. Xu Yingqi, Cheng Yu, and Wang Jingjing [18], on the other hand, believe that in the construction of indicators, pollution to the environment should be considered, and when analyzing the carbon emission efficiency of low-carbon test cities, capital, labor, workforce, etc., are used as inputs. GDP is used as expected outputs, and CO_2 is used as unintended outputs. In terms of research objects, most of the existing literature focuses on industrial enterprises, heavy polluting enterprises, and pollution-intensive industries, such as Zhang Liao and Huang Leiqiong [30], eliminating other external influencing factors to measure the actual green technology innovation efficiency of industrial enterprises, Sun Yanan, Fei Jinhua [31] On the other hand, it is believed that heavy polluting enterprises should pay more attention to green technology innovation, so the creation efficiency of serious polluting enterprises is measured.

Author Copy • Author Copy • Author Copy • Author Copy • Author Copy • Author Copy • Author Copy • Author Copy • Author Copy • Author Copy • Author Copy

Through combing the relevant literature at home and abroad found that the existing research has the following shortcomings: 1) most of the research methodology uses traditional DEA, the efficiency of more than 1 decision-making unit cannot be further compared, and only focus on static efficiency, ignoring the analysis of dynamic efficiency; 2) the research content ignored the problems brought about by environmental pollution and resource depletion, and also ignored the impact of ecological regulatory policies on the efficiency of green technological innovation; 3) In the selection of indicators, scholars have not reached a broad consensus on the choice of needles, and fewer scholars have selected indicators closely related to green technology innovation efficiency; 4) The relevant research objects are concentrated in the enterprise level, and there is a lack of research on other industries, such as high-tech industries. Therefore, based on previous studies, this paper adopts the super-efficiency SBM method, combined with the Malmquist-Luenberge index, to analyze the inter-period dynamic analysis of green technological innovation efficiency, which can more accurately dig into the reasons for the differences; In addition, according to the theory of Porter's hypothesis, in the process of long-term development, the reasonable environmental regulation policy will make enterprises pay more attention to R&D and actively transform into green production enterprises. In addition, according to the theory of Porter's hypothesis, in the long-term development process, reasonable environmental regulation policies will make enterprises pay more attention to R&D, actively transform into green production enterprises, and improve their level of green innovation [32]. Considering that different types of environmental regulation have other effects on the efficiency of green technological innovation, this paper introduces command-and-control, market incentive, and public participation environmental regulatory policies as input indicators; previous scholars have used ordinary patents to indicate the output of innovation results but to reflect "green," this paper, based on the previous research, uses the WIPO Green List to analyze the production of green technology innovation and uses the WIPO Green Inventory as an input indicator. To better reflect the "green," this paper, based on the previous research, uses the WIPO Green List to screen out the green patents, including green invention patents and green utility model patents, as the innovation output. And the entropy value method is used to calculate the comprehensive environmental index of wastewater, exhaust gas, and general waste solids as the non-desired output-related index, which further enriches and improves the green technology innovation index. The high-tech industry is an essential strategic industry in the national economy, so it is crucial to take the high-tech industry as the research object, use the super-efficiency SBM model and Malmquist model, including non-expected output, measure the static efficiency and dynamic efficiency of green technological innovation under the two-phase perspective, analyze the reasons for the differences in each state, and put forward targeted countermeasures, which has a paramount practical significance for the realization of the sustainable development of the economy. This is of great practical relevance for realizing sustainable economic growth.

Measurement Model and Indicator Selection

Measurement Models

Super-Efficient SBM Modeling

The DEA model is a typical representative of the nonparametric method, which is an evaluation method that calculates the relative efficiency by taking the optimal functional equation as a criterion. However, the traditional DEA does not consider the effect of the difference between the respective changes of inputs and outputs in the research object on the efficiency value or whether it is input-oriented or output-oriented. Therefore, Tone [17] has studied the traditional DEA model. Finally, based on the DEA model, it proposes a method model that does not need to consider the differences in the changes of inputs and outputs and does not need to predetermine the dominant direction in advance, i.e., the SBM model. The SBM model breaks the problems of the differences in the changes of inputs and outputs and the selection of the dominant direction of the traditional DEA model. The results are relatively more accurate, and the formula of the SBM model is:

$$\min z = \frac{1-\frac{1}{m}\sum_{i=1}^{m}\frac{s_i^-}{x_{i0}}}{1-\frac{1}{s}\sum_{i=1}^{s}\frac{s_r^+}{y_{r0}}} \tag{1}$$

and subject to the following conditions:

$$st.\begin{cases} x_0 = X\lambda + S^- \\ y_0 = Y\lambda - S^+ \\ \lambda_0 \geq 0, S^- \geq 0, S^+ \geq 0 \end{cases} \tag{2}$$

The value of z in the above Equation (1) denotes the efficiency value of the decision variable; S^+ denotes the slack variable of inputs, S^- denotes the slack variable of outputs, and both of them should be greater than or equal to zero; m denotes the number of inputs; x_{i0} denotes the value of inputs of the decision unit, y_{r0} denotes the value of outputs; s denotes the number of outputs, and s denotes the number of outputs, the number of outputs, and so on. λ denotes the weight vector. Where a value of 1 for the efficiency value z indicates that the decision variable being evaluated is efficient, while

Author Copy • Author Copy • Author Copy • Author Copy • Author Copy • Author Copy • Author Copy • Author Copy • Author Copy • Author Copy • Author Copy • Author Copy

when the value of z is less than 1, the decision variable is inefficient.

When solving the efficiency value according to the SBM model method, the efficiency value of the decision variable is generally less than 1. Still, at the same time, there will be a situation where there are many decision variables in the research object whose efficiency values are all 1, in which case it is not possible to use the efficiency value of the decision variable to determine the efficiency of the research object. It is necessary to use the Super-SBM model, also known as the super-efficiency model, which can further compare the efficiency value of the decision-making unit that has already reached efficiency. The formula of the super-efficiency SBM is as follows:

$$Z^* = \min \frac{\frac{1}{m}\sum_{i=1}^{m}\frac{a_i}{x_{i0}}}{\frac{1}{n+k}\left(\sum_{r=1}^{n}\frac{b_r}{y_{r0}}+\sum_{l=1}^{k}\frac{c_l}{p_{l0}}\right)}$$
$$s.t.\begin{cases} a \geq \sum_{j=1,\neq 0}^{J}\lambda_j x_j \\ b \leq \sum_{j=1,\neq 0}^{J}\lambda_j y_j \\ c \geq \sum_{j=1,\neq 0}^{J}\lambda_j b_j \\ a \geq x_0, b \leq y_0, c \geq p_0, b \geq 0, \lambda_j \geq 0 \end{cases} \tag{3}$$

(3) where m, n, k represents the number of input indicators, desired output, and non-desired output indicators, respectively. x_{i0} denotes the input value, y_{r0} denotes the desired output value, p_{l0} denotes the non-desired output value, j denotes the province and city, a b, and c all represent the slack variables, λ is the weight vector, z* green technology innovation efficiency value, z* the larger, representing the higher level of green technology innovation efficiency.

Malmquist-Luenberger Index

ML indices allow for dynamic analysis of efficiency, and the one used in this paper is the global Malmquist-Luenberger index proposed by Oh [33] et al. This index defines the production frontier of each decision unit in period t as. $P^t(x^t) = \{(y^t, b^t)|x^t$ capable of producing $(y^t, b^t)\}$, $t = 1, 2, ...T$, and the global production frontier as. $P^G(x) = P^1(x^1)\cup P^2(x^2)\cup P^T(x^T)$, where $x = (x_1, x_2, ..., x_N)$, $x\in R^N+$ represents inputs, and $y = (y_1, y_2, ..., y_N)$, $y\in R^M+$ represents desired output, and $b = (b_1, b_2, ..., b_J)$, $b\in R^J+$ represents non-desired output. The direction vector is defined as $g = (g_y, g_b)$, $g\in R^M+ \times R^J$, In this paper, we refer to Y.H., Chung [34] and set the direction vector as $g = (y, b)$, According to the definition of the global production technology set, the ML index of international reference is constructed as follows:

$$ML^{t,t+1} = \frac{1+\vec{D}_0^G(x^t, y^t, b^t; y^t, b^t)}{1+\vec{D}_0^G(x^{t+1}, y^{t+1}, b^{t+1}; y^{t+1}, b^{t+1})} \tag{4}$$

(4) where Eq. $\vec{D}_0^s(x^t, y^t, b^s; y^t, b^s)$ is the directional distance function, indicates the probability that an increase in desired outputs will be accompanied by a decrease in non-desired outputs, the $\vec{D}_0^s(x^s, y^s, b^s; y^s, b^s) = \{\beta:(y^s + \beta^s, b^s - \beta b^s \in P^G(x^s)\}$, $s = t, t+1$, The ML index can be decomposed into the technical efficiency index (EC) and technological progress index (TC):

$$\begin{aligned} ML^{t,t+1} &= \frac{1+\vec{D}_0^G(x^t, y^t, b^t; y^t, b^t)}{1+\vec{D}_0^G(x^{t+1}, y^{t+1}, b^{t+1}; y^{t+1}, b^{t+1})} = \frac{1+\vec{D}_0^t(x^t, y^t, b^t; y^t, b^t)}{1+\vec{D}_0^{t+1}(x^{t+1}, y^{t+1}, b^{t+1}; y^{t+1}, b^{t+1})} \\ &\times \frac{1+\vec{D}_0^G(x^t, y^t, b^t; y^t, b^t)) \Big/ (1+\vec{D}^t(x^t, y^t, b^t; y^t, b^t))}{(1+\vec{D}_0^G(x^{t+1}, y^{t+1}, b^{t+1}; y^{t+1}, b^{t+1})) \Big/ (1+\vec{D}_0^{t+1}(x^{t+1}, y^{t+1}, b^{t+1}; y^{t+1}, b^{t+1}))} \\ &= \frac{TE^t(x^t, y^t, b^t; y^t, b^t)}{TE^{t+1}(x^{t+1}, y^{t+1}, b^{t+1}; y^{t+1}, b^{t+1})} \times \frac{TPG^{G,t}(x^t y^t, b^t; y^t, b^t)}{TPG^{G,t+1}(x^{t+1}, y^{t+1}, b^{t+1}; y^{t+1}, b^{t+1})} \\ &= EC^{t,t+1} \times TC^{t,t+1} \end{aligned} \tag{5}$$

included among these:

$$EC^{t,t+1} = \frac{TE^t(x^t, y^t, b^t; y^t, b^t)}{TE^{t+1}(x^{t+1}, y^{t+1}, b^{t+1}; y^{t+1}, b^{t+1})},$$
$$TC^{t,t+1} = \frac{TPG^{G,t}(x^t, y^t, b^t; y^t, b^t)}{TPG^{G,t+1}(x^{t+1}, y^{t+1}, b^{t+1}; y^{t+1}, b^{t+1})} \tag{6}$$

that is: $ML^{t,t+1} = EC^{t,t+1} \times TC^{t,t+1}$

In the above equation, the TE^s represents the change in production efficiency, and $TPG^{G,S}$ represents the current production frontier of the decision unit P_S and the global production frontier P_G, EC represents the improvement of technical efficiency, when EC>1, it indicates that the technical efficiency has been improved, and vice versa, it will be reduced; TC represents the technological progress; if TC>1, it suggests that there is apparent technical progress, and vice versa, it indicates regression; ML>1 demonstrates that the efficiency of green technological innovation is improving, and vice versa, it means that the efficiency of green technological innovation is decreasing.

Selection and Description of Indicators

Under the perspective of the innovation value chain, the process of innovation activities of enterprises can be further staged, dividing innovation activities into the stage of technology research and development and the location of transformation of scientific and technological achievements, which can explore and discover the efficiency of each step and its changes in a more detailed way – process diagram of two phases of green technology innovation 1 Shown.

Author Copy • Author Copy • Author Copy • Author Copy • Author Copy • Author Copy • Author Copy • Author Copy • Author Copy

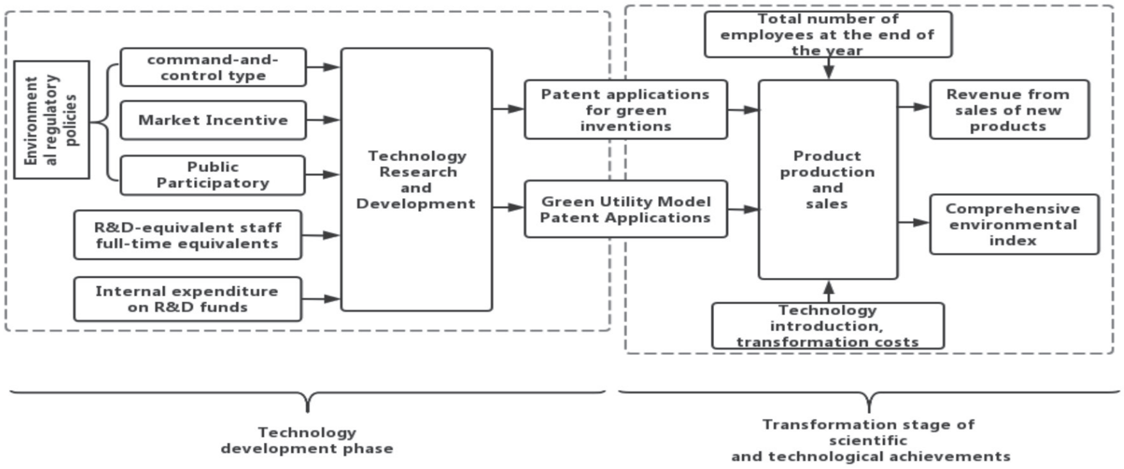

Fig. 1. Two-stage process of green technology innovation.

Input and Output Indicators for the R&D Phase

(1) Input indicators: R&D investment is a decisive factor for enterprises' green innovation output, and R&D expenditure and R&D personnel are commonly used to measure the level of enterprises' R&D investment. Because of the lack of data specializing in green innovation personnel and green R&D expenditure, this paper draws on the practice of Xiao Renqiao, Shen Lu, and Qian Li [35] to express green innovation personnel in terms of R&D personnel converted to full-time equivalents, and green R&D expenditure in terms of internal spending in R&D expenditure. This is mainly due to the difficulty distinguishing between ordinary and green innovators and the fact that the outputs of many non-green technologies also bring some environmental and economic benefits.

When enterprises carry out innovative activities, they are often affected by the government's environmental regulatory policies, and there is no unanimous conclusion on the impact of environmental regulation on green technology innovation, and different types of ecological regulatory policies have other effects on innovation efficiency. In the process of innovation activities, the rules issued by the government have a solid binding impact on enterprises, forcing them to improve in the direction of green technology and reduce environmental pollution behaviors; therefore, this paper draws on the approaches of Li Bin, Milani S, et al. [36, 37] scholars, considering that regulations have a continuous impact, the number of local environmental protection laws, regulations, and standards are accumulated year by year to represent the command-and-control environmental regulation; as the public becomes more and more concerned about environmental issues, the government and enterprises will take the lead in assuming the responsibility of environmental protection, which will lead to a better public image and lower social governance costs. Therefore, this paper draws on the practice of Shim Shack J.P., Ward M.B. [38] and uses the number of sudden environmental events to represent the public participation type of environmental regulation. The number of sudden environmental events refers to the number of ecological events involving public safety that occur suddenly, cause or are likely to cause significant casualties, major property damage, and pose a considerable threat and damage to the economic and social stability and political stability of the whole country or a specific region, and have a significant social impact. In addition, Testa, Iraldo, and Frey [39] found that economic incentive-based environmental regulation means will reduce the financial profit of enterprises, market incentive-based environmental regulation requires enterprises to pay for the purchase of sewage rights, which is an additional cost burden on enterprises. It will have a crowding-out effect on the green development of enterprises. Hence, this paper adopts the amount of sewage charge levy to indicate the market incentive-based environmental regulation.

Output Indicator: In the R&D stage, enterprises invest green personnel and capital, and their outputs are mainly in the form of patents and other scientific and technological knowledge. The number of patent applications can better reflect the actual performance of enterprises in technological innovation. Among all patents, invention, and new-type utility patents are characterized by solid innovativeness and high social recognition, representing an enterprise's scientific and technical output capacity. This paper selects the number of green invention patent applications and green utility model patent applications as output indicators [40]

.

Input and Output Indicators for the Transformation Phase

(1) Input indicators: Technological transformation is how an enterprise transforms the scientific and technical results developed into tangible assets. The input indicators of this stage include innovative inputs and non-innovative inputs. Creative inputs are represented by the outputs of the R&D stage, i.e., patents because in the two-stage perspective, the outcomes do not

Author Copy • Author Copy • Author Copy • Author Copy • Author Copy • Author Copy • Author Copy • Author Copy • Author Copy • Author Copy • Author Copy

immediately exit the enterprise innovation system but continue to contribute to the innovation process as a new input. In addition, using patents as an indicator of information in the technology transformation stage is enough to verify the extent to which patents are transformed in the innovation system and whether the socio-economic benefits, they bring are favorable. In addition, to transform patents into practical economic value, some non-innovative inputs need to be added, such as the introduction fee of technological transformation and participating personnel. Therefore, based on the formation of green patents by green technological innovation, this paper draws on the practice of Wu Zenghai and Li Tao [41] and selects technological transformation, introduction fees, and end-of-year employees to represent the non-innovative inputs for technological change in this industry.

(2) Output indicators: The goal of green technology innovation is to realize economic growth and environmental quality improvement at the same time. Hence, this paper selects the expected results in terms of economy - new product sales revenue, and the unintended results in terms of environmental benefits - the comprehensive environmental index Therefore, this paper selects the expected result in terms of economy - new product sales revenue, and the unanticipated consequence in terms of ecological benefit - comprehensive environmental index, which are used to measure the result of green technology innovation. Among them, the new product sales revenue is the economic performance of the enterprise's green innovation results, which can reflect the financial contribution of green technology to the enterprise. The comprehensive environmental index is a one-dimensional measure of industrial wastewater emissions, industrial sulfur dioxide emissions, and general waste solid disposal using the entropy method. Overall, the lower the emissions of these wastewater, waste gas, and solid waste, the higher the efficiency of the enterprise's green technology innovation. The more it can reflect the eco-efficiency of that innovation project [11]. The system of green invention two-stage indicators is shown in Table 1. The two-stage indicator system of green innovation is shown in Table 1.

Data Sources

In this paper, panel data of input and output indicators of high-tech industries in 30 provinces (autonomous regions and municipalities directly under the central government) (excluding Tibet region and Hong Kong, Macao, and Taiwan region due to missing data) from 2011 to 2020 are selected to measure the efficiency of green technological innovation in high-tech industries. And the 30 provinces are categorized into three major regions: east, central, and west, as explained by the National Development and Reform Commission (NDRC). The data of the technology R&D stage and the transformation of the scientific and technological achievements stage are mainly obtained from the China Science and Technology Statistical Yearbook, China Environmental Statistical Yearbook, and the statistical yearbooks of each city in the corresponding years for supplementation, and the intermediate output indicators, green invention patent applications, and green utility model patent applications are obtained from the CnOpenData database.

Measurement Results and Analysis

Static Efficiency

Based on the panel data, China's high-tech industries' green technology innovation efficiency was measured, and its characteristics and change trends were analyzed.

Table 1. Two-stage indicator system for green technology Innovation rate.

Point	Form	Interpretation of indicators
Technology development phase	Manpower inputs	Full-time equivalent of R&D personnel (person-years)
	Capital investment	Internal expenditures on R&D funds (in millions of dollars)
	Environmental regulatory policies	Command-and-control environmental regulation (number of local environmental laws, regulations, standards)
		Market incentive-based environmental regulation (amount of sewage charges levied)
		Public participatory environmental regulation (number of environmental emergencies)
	Intermediate output indicators	Patent applications for green inventions (pieces)
		Green utility model patent applications (pieces)
Transformation stage of scientific and technological achievements	Manpower inputs	Number of employees at the end of the year (person-years)
	Capital investment	Technology introduction and renovation costs (in millions of dollars)
	Expected outputs	Revenue from sales of new products (in millions of dollars)
	Non-expected outputs	Composite environmental index (%)

Author Copy • Author Copy • Author Copy • Author Copy • Author Copy • Author Copy • Author Copy • Author Copy • Author Copy • Author Copy

The results are shown in Table 2. In terms of the mean value, the mean value of green technology innovation efficiency of China's high-tech industries increased from 0.4688 in 2011 to 0.5740 in 2020, showing a relatively small increase; the national mean value was 0.5277, and during the ten years, there were six years in which the efficiency value was higher than the national mean value and four years in which the efficiency value was lower than the national mean value; the efficiency value of provinces was classified into different grades by using the natural breakpoint method [42]: Low efficiency (0~0.4), medium-low efficiency (0.4~0.6), medium-high efficiency (0.6~0.8), and high-efficiency (more than 0.8). Among the 30 provinces, there are far more low-efficiency provinces (21) than high-efficiency provinces (9), which shows that China's green technological innovation in high-tech industries is overall lower, and there are significant differences between areas. In the

Table 2. Green technology innovation efficiency of China's high-tech industries, 2011-2020.

Area	2011	2012	2013	2014	2015	2016	2017	2018	2019	2020	Average value	Rankings
Guangdong	1.5698	1.5346	1.4800	1.4285	1.3402	1.3845	1.4895	1.4859	1.5432	1.5149	1.4771	1
Tianjin	1.1743	1.0904	1.5946	1.2384	1.2724	1.3761	0.7444	1.0175	0.6128	1.1437	1.1265	2
Beijing	1.6504	1.3237	1.3856	0.7415	0.6867	0.6754	1.0564	1.1726	1.2529	1.2751	1.1220	3
Henan	0.1288	0.1693	1.4820	1.3342	1.3212	1.4039	1.3445	1.4804	1.1974	1.2892	1.1151	4
Jiangsu	1.0678	1.3316	1.0563	1.1534	1.1213	1.1045	0.7723	1.1253	1.1413	1.2573	1.1131	5
Sichuan	0.4240	0.2317	0.8789	0.3588	1.1123	0.8508	0.8484	0.7680	0.7986	0.3967	0.6668	6
Gansu	0.2470	0.4003	1.0666	0.1596	1.1445	0.3488	1.1691	0.3799	1.0008	0.5998	0.6516	7
Shanxi	0.1108	0.2573	0.4895	0.4324	1.0532	0.8013	0.7280	0.7106	0.7818	0.7318	0.6097	8
Chongqing	0.6341	0.2619	0.1886	0.4174	0.8028	0.7048	0.8129	0.6937	0.6378	0.7883	0.5942	9
Hebei	0.7277	0.8332	0.8353	1.1670	0.3873	0.4186	0.2820	0.2540	0.4839	0.4613	0.5850	10
Hainan	1.0853	1.1934	0.3631	0.4615	0.4283	0.2886	0.6516	1.0238	0.1142	0.1254	0.5735	11
Shanghai	0.4449	0.3923	0.3301	0.4343	0.5461	0.6800	0.5747	0.6912	0.6258	0.7091	0.5428	12
Hubei	0.2602	0.3363	0.4224	0.2274	0.4404	0.5733	0.7457	0.7438	0.7585	0.6632	0.5171	13
Shandong	0.6327	0.5572	0.7401	0.5995	0.5510	0.4282	0.3798	0.4797	0.3593	0.3811	0.5109	14
Zhejiang	0.2625	0.2661	0.3441	0.3080	0.3933	0.3435	0.5480	0.7058	0.9053	0.8274	0.4904	15
Jiangxi	0.1447	0.1216	0.2005	0.3185	0.2559	0.6234	0.5623	1.0204	0.7917	0.6992	0.4738	16
Fujian	0.4601	0.4734	0.3677	0.3656	0.3717	0.3223	0.3000	0.4347	0.5051	0.5864	0.4187	17
Liaoning	0.6225	0.3002	0.4336	0.5170	0.7326	0.3111	0.2955	0.3451	0.2882	0.2791	0.4125	18
Jilin	0.1881	0.2236	0.4438	0.5108	0.3541	0.2978	0.4402	0.3027	0.6617	0.4164	0.3839	19
Guangxi	0.1023	1	0.2718	0.2335	0.3029	0.2084	0.3512	0.5382	0.5325	0.2858	0.3827	20
Anhui	0.1883	0.2211	0.2434	0.2704	0.3584	0.4279	0.3891	0.4465	0.4011	0.4983	0.3444	21
Qinghai	0.6036	0.2391	0.3141	0.3065	0.3374	0.2929	0.3105	0.3050	0.3008	0.3105	0.3320	22
Hunan	0.2080	0.2351	0.3233	0.3039	0.2738	0.2840	0.2533	0.2955	0.3147	0.3951	0.2887	23
Yunnan	0.4669	0.3429	0.2315	0.1819	0.1304	0.1573	0.1620	0.1777	0.3588	0.5937	0.2803	24
Shaanxi	0.1716	0.1209	0.1202	0.1175	0.1446	0.1928	0.2092	0.2226	0.2620	0.2278	0.1789	25
Heilongjiang	0.0543	0.0808	0.1173	0.1254	0.1781	0.1340	0.1469	0.1783	0.3632	0.2363	0.1615	26
Guizhou	0.1099	0.1149	0.1311	0.1491	0.1242	0.1931	0.1599	0.2277	0.1682	0.1641	0.1542	27
Mongolia	0.1274	0.1839	0.1226	0.1300	0.1304	0.1128	0.1389	0.1155	0.1212	0.1086	0.1291	28
Xinjiang	0.0332	0.0008	0.1312	0.1114	0.1032	0.0367	0.1217	0.1366	0.1058	0.1926	0.0973	29
Ningxia	0.1018	0.1260	0.1011	0.1010	0.1047	0.0635	0.1002	0.1022	0.1014	0.0619	0.0964	30
Average value	0.4668	0.4655	0.5403	0.4735	0.5501	0.5013	0.5363	0.5860	0.5830	0.5740	0.5277	

Author Copy • Author Copy • Author Copy • Author Copy • Author Copy • Author Copy • Author Copy • Author Copy • Author Copy • Author Copy • Author Copy

future, the focus will be on improving efficiency and the coordinated development of regions. In the end, the focus is not only on how to improve efficiency but also on regional coordinated development. From the inter-provincial level, Guangdong Province (1.4771) and Tianjin Municipality (1.1265) are the two regions with the best green technological innovation efficiency. Among them, the green technical innovation efficiency of Guangdong Province only slightly decreased from 2013 to 2016 and then steadily rebounded, indicating that the structure of inputs and outputs in Guangdong Province has gradually become reasonable; Tianjin Municipality has only had an efficiency of less than 1 in two of the ten years, indicating that in these ten years, the efficiency was less than 1 in the past ten years. This demonstrates that Tianjin can make better use of resources and maintain the unity of economic development and environmental protection in the process of growth during this decade and that Tianjin, as a first-tier city in China, with a high degree of marketization, abundant talent, and perfect technological development and supporting facilities, is a favored place for hi-tech enterprises, which pay great attention to both scientific and technical R&D inputs as well as technological transformations; followed by Beijing, Jiangsu, and Henan, etc. Secondly, Beijing, Jiangsu, and Henan rank high in green innovation efficiency. The average value of efficiency exceeds the national average, which shows that the level of green technology and enterprise management in these provinces is better, among which Beijing and Jiangsu are economically developed regions in China, which are also the concentration of talents and science and technology, with a perfect governance system and an optimized innovation environment, such as Beijing, which has already explored a synergistic development path of green development and scientific and technological innovation and has taken advantage of its strengths to attract talents extensively.

Jiangsu Province, as a significant manufacturing province in China, has continuously strengthened digital green transformation, accelerated the technological innovation of green change, and strengthened the synergistic cooperation between enterprises and the manufacturing industry so that the efficiency of green technological innovation has been maintained at a high level. Henan is located in the middle and lower reaches of the Yellow River. It is an essential center of the nation's people, logistics, and information flow, and it can flexibly adjust the direction of technological development during the enterprise development process; however, its neighboring provinces, such as Shaanxi, have poor outcomes of green technological innovation. However, its adjacent regions, such as Shaanxi, could be doing better in developing green technological innovation. This shows that Henan has yet to give full play to its leading role and to radiate to its neighboring provinces. It should accelerate the formation of a continuous development trend and agglomeration development effect while developing itself in the future. The lower rankings are Ningxia, Xinjiang, Inner Mongolia, Guizhou Province, Heilongjiang Province, etc. These areas are remote, lack scientific and technological talents, are relatively backward in terms of education and economic level, and the foundation of the green technology industry needs to be stronger, thus bringing poor returns on technological inputs.

The curves in Fig. 2 reflect the efficiency values of the eastern central and western regions at different times. An efficiency value above 1 indicates that the region's green technology innovation efficiency is effective. The average value of green technology innovation efficiency in the eastern region is 0.7782, leading the other two regions. Overall, the green technology innovation efficiency is highest in the east, followed by the center. Finally, the west and the three regions show a spatial distribution pattern that decreases from the east to the center to the west. From the perspective of each region, the green technology innovation efficiency curve of high-tech industries in the eastern region can be roughly divided into two segments, the first segment is from 2011 to 2017, the innovation efficiency fell from 0.8816 to 0.6449, a decline of 36.8%, the curve shows a downward trend year by year, and the second segment is after 2017, it shows a slow and unstable rebound, and the value of the efficiency in 2020 rebounded to 0.7782, which It shows that the input-output efficiency of the eastern region gradually tends to stabilize; the central region shows the characteristics of rapid and stable rise, from 0.2056 in 2011 to 0.6162 in 2020, rising faster than the other two regions, especially after 2017, the efficiency value of its green technological innovation and the east has not much difference, which shows that the central region has received extensive attention in recent years, and can use the government regulation policies, rationally arrange the input of resource elements, maximize the use of their own advantages, and constantly approach the direction of resource protection and economic development; the western region has experienced many rises and declines, showing a wavy trend of change, with a small increase in the efficiency value during the 10-year period, from 0.4081 in 2011 to 0.4505 in 2020, and reaching a peak in 2017 (0.5020), the unstable green technology innovation efficiency means that this region needs to be focused on in the future; overall, although the efficiency of the central and western regions is lower than that of the east, the growth of both is larger than that of the east, and after the development in recent years, the level of green technology innovation in the central and western regions is constantly approaching the east, which indicates that there is a huge potential for high-tech industries in the central and western regions in improving the efficiency of green innovation, especially in the central region, which has been gradually (Note: According to the general classification method, the level of green technological innovation in the central and western regions has been converging with that in the east. (Note: According to the general

Author Copy • Author Copy • Author Copy • Author Copy • Author Copy • Author Copy • Author Copy • Author Copy • Author Copy

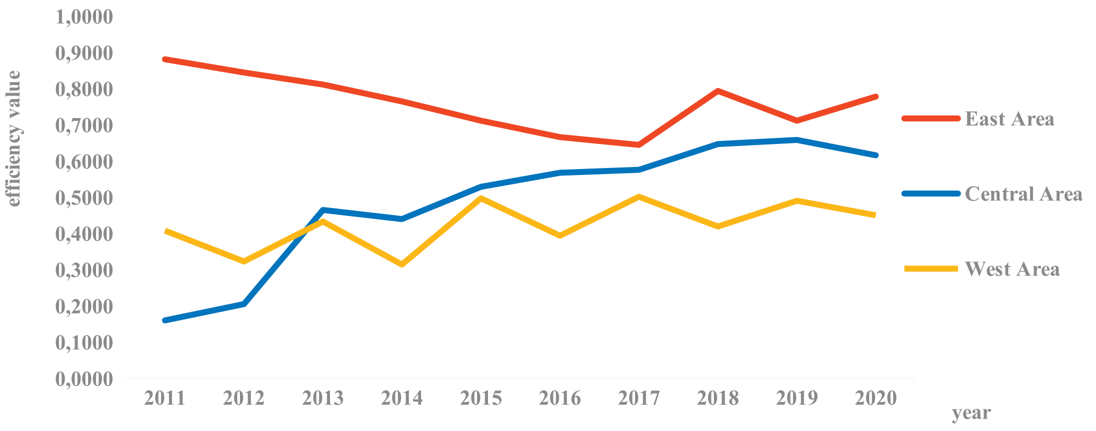

Fig. 2. Trends in green technology innovation in high-tech industries in three major regions, 2011-2020.

division method, the east includes Beijing, Liaoning, Tianjin, Hebei, Shanghai, Jiangsu, Zhejiang, Fujian, Shandong, Guangdong, and Hainan; the center includes Shanxi, Anhui, Jiangxi, Henan, Hubei, Hunan, Jilin, and Heilongjiang; and the west includes Inner Mongolia, Guangxi, Chongqing, Sichuan, Guizhou, Yunnan, Tibet, Shaanxi, Gansu, Qinghai, Ningxia, and Xinjiang)

According to the results of existing research, considering the official implementation of the new environmental law in 2015, 2016 is the first year of the acceptance of the results of the environmental protection law, in order to be more able to reflect the current stage and future trends of the regions, the data of 2016 and 2020, the last year of the examination period, were selected for comparison, see Table 3, in the period of the examination, Guangdong Province and Henan Province have been steadily ranked in the high-efficiency ranks in the last five years, Jiangsu Province and Tianjin Province are located in the high-efficiency row column in the rest of the years except for 2017 in the medium-high-efficiency row; Beijing has risen from the medium-high-efficiency row in 2016 to the high-efficiency row in 2021, which can be seen that Beijing has permanently attached importance to the green technological innovation; Zhejiang Province has risen from the low-efficiency to the high-efficiency row in the five-year period, and its development is remarkably rapid, because in recent years, Zhejiang Province is vigorously encouraging the research and development of green technology and actively exploring the "Internet + energy saving and environmental protection" new model, and constantly improve the policy guidance mechanism, vigorously promote the green development of the manufacturing industry, the number of patents on green technology has increased year by year, only located in Jiangsu, Beijing and Guangdong after. Jiangxi, Shanghai, and Chongqing are consistently among medium and high-efficiency ranks. The stability of their green innovations cannot be separated from the support and guidance of policies, such as during the 13th Five-Year Plan period, Jiangxi Province has continuously improved the subsidy mechanism for clean electricity and then increased the subsidy standard for new energy vehicles; Hubei has been upgraded from medium and low efficiency to medium and high efficiency, and the number of provinces belonging to the medium and high efficiency has been changed from four to five. Most of the regions with high and medium efficiency originate from the east and center of the country, which is consistent with the previously obtained conclusion that the east and center of the country are relatively more efficient.

From 2016 to 2020, there was a gradual increase in the number of medium and low-efficiency provinces, from 4 to 6, of which Fujian, Gansu, Jilin, Yunnan, and other regions have risen from the previous low-efficiency to medium and low-efficiency, indicating that the overall efficiency of green technological innovation of China's high-tech industries has been improved by a small margin in the period under investigation. The number of inefficient provinces has decreased by three during the five years. The low-efficiency sections mainly originated from the less-developed regions in the west, of which Guizhou, Qinghai, Guangxi, Xinjiang, Inner Mongolia, and other places have been in the low-efficiency areas. However, these places have superior ecological environments. The environmental protection and the pollution caused by the industrial process are contradictory to this problem but also need to be solved, such as Guizhou, a large coal mining province in the west, in the process of exploiting many mineral resources, caused severe pollution of the environment, resulting in green technological innovation is not efficient, the previous section can explain this analysis. This analysis can explain the specific reasons for the low efficiency of the western region obtained in the last area; Heilongjiang Province located in the northeastern area of Liaoning Province has also been in the low efficiency level, a possible reason is that as the main heavy industrial base of the country, the northeastern region gives priority to the development of heavy industry, making a significant contribution to the economy, at the same time, high energy-consuming and high-polluting industries to the environment has brought about a great deal of pressure, along with the reform

Table 3. Distribution of Green Technology Innovation Efficiency Levels.

Particular year	High efficiency	Medium-to-high efficiency	Medium to low efficiency	Inefficiency
2016	Guangdong, Henan, Jiangsu, Shanxi, Sichuan, Tianjin	Beijing, Jiangxi, Shanghai, Chongqing	Anhui, Hebei, Hubei, Shandong	Fujian, Gansu, Guangxi, Guizhou, Hainan, Heilongjiang, Hunan, Jilin, Liaoning, Inner Mongolia, Ningxia, Qinghai, Shaanxi, Xinjiang, Yunnan, Zhejiang
2020	Beijing, Guangdong, Henan, Jiangsu, Tianjin, Zhejiang	Hubei, Jiangxi, Shanxi, Shanghai, Chongqing	Anhui, Fujian, Gansu, Hebei, Jilin, Yunnan	Guangxi, Guizhou, Hainan, Hunan, Heilongjiang, Liaoning, Inner Mongolia, Ningxia, Qinghai, Shandong, Shaanxi, Sichuan, Xinjiang

of the economic system and the transformation of the industrial structure, the investment structure of the With the reform of the financial system and the change of industrial form, the distortion of the investment structure in the post-industrial era of the drawbacks are more prominent, and its level of economic development is more and more lagging behind, which in turn leads to a large number of talent outflow. Northeast China should seize the opportunity of the national revitalization of the old industrial base in Northeast China, quickly adjust the industrial structure, optimize the scientific research system, relax political authority, encourage local research institutes to research and development of green technology, retain local well-known and efficient talents, and continue to absorb innovative skills from all over the country, to achieve historic innovation. In conclusion, due to the large scale of China's territory, the economy, geographic environment, and historical factors leading to the unbalanced development between provinces and cities [43], the performance of green technology innovation efficiency between regions is not the same; therefore, to make the balanced and coordinated development of the provinces and reduce the differences between the sections is the direction of China's efforts in the future.

Fig. 3 reflects the changes in the efficiency of the technology R&D stage, the efficiency of the science and technology achievement conversion stage, and the overall efficiency between 2011 and 2020. First, the efficiency of the technology R&D stage reached its highest in 2013 and then showed a wave-like increasing and decreasing state, and the efficiency value is greater than 1 in all the years except for 2016 and 2020, which are less than 1. The decrease and increase in the scientific and technological achievements conversion stage are smaller, but the increase is more significant than the decrease, so the overall shows a wave-like upward trend; comparing the curves of the two phases, we can see that the input and output efficiency of the technology R&D stage is higher than the Input and output efficiency of the stage of transformation of scientific and technological achievements. In the examination period, the gap between the efficiency levels of the two phases is gradually narrowing and converging to a consistent state. Secondly, from the figure, we can see that the fluctuation trend of the overall efficiency curve and the fluctuation direction and amplitude of the angle of the transformation stage of scientific and technological achievements are almost the same, which can be seen that the transformation stage of scientific and technical achievements has a more significant impact on the overall efficiency. The difference between the two efficiency values is optional, indicating that we should maintain the advantages of research and development in innovation activities and pay more attention to synergizing the relationship between the two stages of efficiency transformation. In conclusion, the analysis, on the one

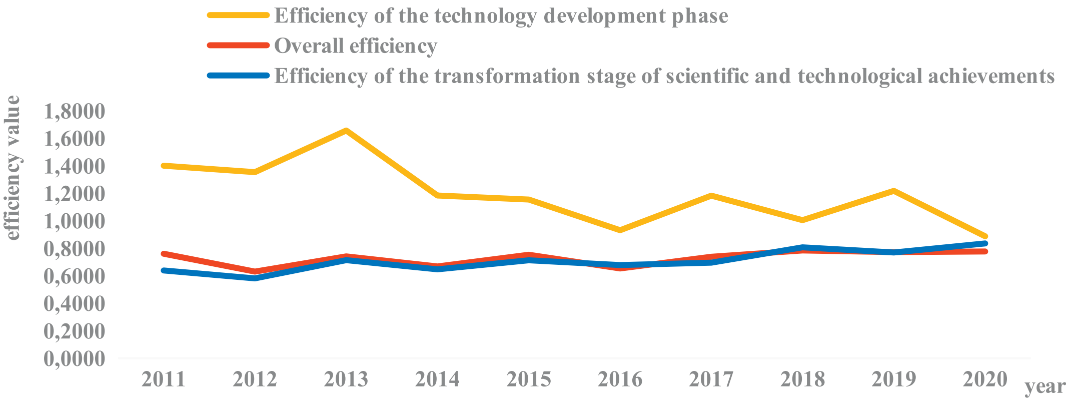

Fig. 3. Trend of green technology innovation efficiency and overall efficiency in two stages.

Author Copy • Author Copy • Author Copy • Author Copy • Author Copy • Author Copy • Author Copy • Author Copy • Author Copy

Author Copy • Author Copy • Author Copy • Author Copy • Author Copy • Author Copy • Author Copy • Author Copy • Author Copy

hand, shows that in China's high-tech industry, green technology innovation two-stage efficiency level, there is still ample space for development; on the other hand, can be seen that the two stages of efficiency together affect the overall efficiency of the change, therefore, should maintain the R & D stage of the efficiency of the advantages of the high efficiency at the same time, pay more attention to scientific and technological achievements into the efficiency of the enhancement of the efficiency.

Dynamic Analysis of the Malmquist-Luenberger Index

The dynamics of green technology innovation can be well reflected by the Malmquist-Luenberger index [44], and its decomposition value can be used to explore the deep-rooted reasons affecting the changes in the ML index, see Table 4.

From a general point of view, the average value of China's ML index reaches 1.0719, which indicates that China's green technological innovation level is in a state of progress; among the 30 provinces, 19 provinces have an ML index greater than 1, accounting for 63.3%, of which 16 areas have an ML index more significant than the average value, accounting for 53.3%, indicating that the overall change in the efficiency of green technological innovation in China's high-tech industries in the various provinces is better. However, due to the significant differences in the spatial layout, economic development level, emphasis on policy and technology level of each section, the inter-provincial changes in innovation efficiency are also substantial; further analyzing the decomposition value of ML, it can be seen that there are 22 provinces with a technical efficiency index greater than 1, and there are only eight provinces with a technological progress index greater than 1, which indicates that most of the provinces pay more attention to the improvement of technical efficiency, and neglect the technological progress on the impact of green technological innovation.

The regions with more obvious growth in the ML index include Henan and Jilin, and Jiangxi, indicating that these three regions have been exploring how to allocate resources, manage personnel, optimize the whole innovation process, and have invested a lot of human resources and material resources in the past ten years, and have achieved corresponding benefits and reduced non-expected outputs; the ML index of Sichuan, Tianjin, Chongqing, and Shanghai also grew faster, indicating that the overall development trend of these provinces has been good. The ML index of Sichuan, Tianjin, Chongqing, and Shanghai also grows faster, meaning that the general development trend of these provinces is good, which may be due to the favorable geographic locations of Sichuan and Chongqing and the concentration of scientific and technological talents in Tianjin in recent years, which should continue to maintain a fast and stable development in the future. In addition, the ML index of Zhejiang, Anhui, Hubei, and Shanxi reaches more than 1, indicating that reforming the science and technology management system in these areas has been effective. After the ML decomposition, the index of technological efficiency in these places is greater than 1, but the index of technological progress change is less than 1. These provinces should take advantage of their strengths and, based on stably making progress, lay out the structure of inputs and outputs more efficiently and reasonably and invest more human resources into innovation management. Human resources into the innovation management process to promote technological progress while keeping the characteristics of technological efficiency growth in good play. Guangxi and Guangdong's ML indexes have also reached more than 10%. Still, the EC is less than 1, thus hindering the improvement of the ML index, and these two places should focus on strengthening the improvement of technical efficiency. The growth of technical efficiency and technical progress indexes in Jiangsu and Beijing are relatively balanced, and both positively promote the advancement of green technology innovation efficiency. The provinces with an ML index less than 1 are Liaoning, Yunnan, Inner Mongolia, Hebei, Qinghai, Shandong, Ningxia, Hainan, Heilongjiang, Xinjiang, Gansu, and other 11 provinces, which can be seen through the ML decomposition, among which, the provinces of Yunnan, Inner Mongolia, Hainan, and Heilongjiang have EC more significant than 1. The TC is less than 1, which indicates that the ML index of these provinces is less than 1, mainly because the change in technological progress is too low. Even if technological efficiency is effective, technical progress negatively affects its effectiveness. The two decomposition values of the ML index of the remaining provinces are all less than 1. The improvement of the ML index of these provinces is not only affected by the decline of technological progress but also negatively affected by technological efficiency, so it can be learned that the input-output layout of these provinces is not too reasonable, and the investment in the cultivation of high-end innovative talents and innovative technology is not enough.

Fig. 4 reflects the ML index and its decomposition value in the east, center, and West. As can be seen from the figure, the growth of the ML index in the eastern region mainly relies on the development of technical efficiency (EC), which is similar to the conclusions of most scholars [45], indicating that the management concepts and technological input conditions in the eastern region are more compatible with the size of the area, such as Beijing and Shanghai located in the east part of the country, which have a large number of renowned colleges and universities and scientific research institutes that constantly contribute to the green technological innovation; at the same time, the central region's ML index growth also relies mainly on the addition of technical efficiency (EC) progress, the efficiency of green technological innovation has been

Author Copy • Author Copy • Author Copy • Author Copy • Author Copy • Author Copy • Author Copy • Author Copy • Author Copy • Author Copy

Table 4. Malmquist-Luenberger index and its decomposition of green technology innovation efficiency in China's high-tech industries.

Area	ML	Rankings	EC	Rankings	TC	Rankings	Area	ML	Rankings	EC	Rankings	TC	Rankings
Henan	1.8140	1	1.8849	1	0.9624	17	Shaanxi	1.0172	17	1.0485	16	0.9702	13
Jilin	1.4099	2	1.0934	11	1.2895	1	Fujian	1.0022	18	1.0429	18	0.9610	18
Jiangxi	1.2641	3	1.2992	3	0.9730	12	Guangdong	1.0021	19	0.9969	23	1.0052	7
Sichuan	1.1948	4	1.3391	2	0.8923	24	Liaoning	0.9829	20	0.9869	24	0.9960	9
Tianjin	1.1750	5	1.0725	14	1.0956	3	Yunnan	0.9367	21	1.1021	10	0.8499	27
Chongqing	1.1527	6	1.1455	7	1.0063	6	Inner Mongolia	0.9343	22	1.0052	22	0.9294	21
Shanghai	1.1330	7	1.0698	15	1.0591	4	Hebei	0.9069	23	1.0434	17	0.8692	26
Hubei	1.1305	8	1.1686	5	0.9673	14	Qinghai	0.8746	24	0.9684	27	0.9031	23
Shanxi	1.1177	9	1.1261	8	0.9925	10	Shandong	0.8719	25	0.9639	28	0.9046	22
Zhejiang	1.1065	10	1.1591	6	0.9546	19	Ningxia	0.8675	26	0.9869	25	0.8790	25
Anhui	1.0856	11	1.1226	9	0.9670	15	Hainan	0.8336	27	1.0351	20	0.8054	28
Guangxi	1.0767	12	0.9819	26	1.0965	2	Heilongjiang	0.6342	28	1.2420	4	0.5107	30
Guizhou	1.0614	13	1.0753	13	0.9871	11	Xinjiang	0.5148	29	0.8822	29	0.5835	29
Hunan	1.0465	14	1.0847	12	0.9648	16	Gansu	0.3236	30	0.3470	30	0.9327	20
Jiangsu	1.0451	15	1.0402	19	1.0047	8	average value	1.0179		1.0773		0.9442	
Beijing	1.0208	16	1.0060	21	1.0148	5							

Author Copy • Author Copy • Author Copy • Author Copy • Author Copy • Author Copy • Author Copy • Author Copy • Author Copy

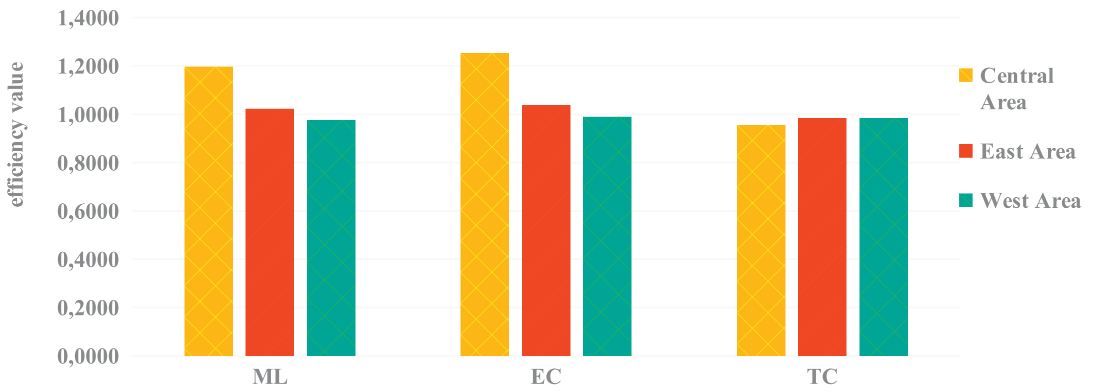

Fig. 4. Malmquist-Luenberger index and its decomposition values for the three regions.

optimized to the greatest extent possible, and even if technological progress is in decline, innovation efficiency is still growing. The probable reason is that with the development of the industrial structure adjustment accelerated, high-tech industries began to transfer to the central region. The industrial cluster effect makes the main part of high-tech industry technology green technology innovation level has dramatically improved, and the technical efficiency index significantly improved; In contrast, the western region ML index is subjected to the negative impact of the decline of technical efficiency (EC) and technological progress (TC), which makes the region ML index Only 0.9755; the western part is located in the remote area, resources, and energy are relatively limited, educational conditions, economic development model is relatively backward, the lack of scientific and technological innovation talents, technological innovation consciousness is not high, its innovation environment and resource conditions are relatively backward, making the development of green technological innovation slow. Overall, the best change in green technological innovation is in the central region, but the technological progress index in the main area is the lowest; the reason for this phenomenon is that the effect of the increase in technical efficiency in the central region offsets the impact of the decline in technological progress; in green technological innovation, the increase in technological efficiency (EC) is an essential factor to promote its role. The middle east and west decrease characterize the ML and EC indexes.

As can be seen from Table 5, from 2011 to 2014, the ML index of the R&D stage was less than 1. From 2014 to 2019, the ML index of the technology R&D stage was more significant than 1, of which the growth rate of the ML index reached more than 10% in 2014-2016 and the growth rate got more than 20% in 2016-2018, which indicates that the efficiency of the R&D stage of China's high-tech industry has been continuous improvement, and the efficiency of the R&D stage may be reduced from 2019 to 2020 due to the impact of the epidemic; the efficiency of the transformation of scientific and technological achievements during the ten years is only occasionally greater than 1 in recent years, which, on the one hand, indicates that the efficiency of the transformation of scientific and technological achievements in China is generally low, which is also a cause of the low efficiency of the overall green technological innovation.

Table 5. Two-stage ML and its decomposition values.

Particular year	Technology development phase			Transformation stage of scientific and technological achievements		
	ML	TC	EC	ML	TC	EC
2011-2012	0.9190	0.9343	0.9836	0.7979	0.8686	0.9187
2012-2013	0.8949	0.9775	0.9155	0.9341	0.7366	1.2680
2013-2014	0.8975	1.0232	0.8772	0.8823	0.9243	0.9546
2014-2015	1.1618	0.9264	1.2540	0.9993	0.8368	1.1942
2015-2016	1.1130	0.9123	1.2201	0.8261	0.8132	1.0158
2016-2017	1.2126	1.1778	1.0295	0.9294	0.9357	0.9933
2017-2018	1.2364	0.9556	1.2939	1.0311	0.8528	1.2091
2018-2019	1.0041	1.0084	0.9957	0.9910	0.9627	1.0294
2019-2020	0.4371	0.8470	0.5161	1.5124	1.4554	1.0392

Author Copy • Author Copy • Author Copy • Author Copy • Author Copy • Author Copy • Author Copy • Author Copy • Author Copy • Author Copy

On the other hand, the high-tech industry has continuously improved the efficiency of the achievements transformation and has seen the first results in recent years. From the perspective of efficiency decomposition, the R&D stage has TC and EC less than 1 in 2011-2013, and EC is greater than 1 in 2014-2018, while TC is greater than 1 in only one year during this period, indicating that the technology R&D stage mainly relies on the improvement of technical efficiency, and technological progress plays a minor role; during the period of examination, the results transformation stage has TC greater than 1 in 2012-2013, 2014- 2016, and 2017-2020 all have EC more remarkable than 1, while TC is only greater than 1 in 2019-2020. The growth of efficiency in the stage of transformation of scientific and technological achievements also relies mainly on the growth of the EC index. Vertically, during the examination period, the ML index of China's high-tech industry's technology research and development stage is generally growing. The ML index of the transformation of scientific and technological achievements step still needs to be more effective. Still, it is also improving, and the efficiency change of the technology research and development stage is higher than the efficiency change of the transformation of the scientific and technological achievements stage. The gap between the efficiency of the two phases is decreasing.

Conclusions and Recommendations

The static and dynamic analysis of the green technology innovation efficiency of China's high-tech industries has led to the following preliminary conclusions:

(1) Although China's green technological innovation efficiency shows a positive trend, the gap between provinces is large, and the overall efficiency is not high, and there is still a lot of room for improvement in the future; (2) The green technological innovation efficiency of the eastern, central and western regions is ranked from high to low, and the differences among the three major regions are not only reflected in the spatial pattern, but also in the growth rate, and the central region is growing particularly fast; (3) China's green technological innovation inefficient provinces are gradually decreasing, indicating that the level of green technological innovation in China's high-tech industries is constantly improving, and the inter-provincial differences are constantly shrinking; (4) During the investigation period, the ML mean value of the green technological innovation efficiency of China's high-tech industries is greater than 1, indicating that its overall development is in a state of progress; (5) From the viewpoint of the three major regions, the improvement of the ML index in the eastern and central parts of China is mainly due to the increase in technological efficiency, and the (5) From the perspective of the three regions, the increase of ML index in the east and center of China is mainly due to the improvement of technical efficiency, while the decrease of ML index in the west of China is affected by the joint decline of technological progress and technological efficiency; (6) From the perspective of the two-stage perspective, the efficiency of the technology research and development stage is higher than the efficiency of the stage of transformation of scientific and technological achievements, and the inefficiency of the stage of transformation of achievements is mainly affected by the inhibition of technological progress. The above conclusions imply the following insights:

(1) Different provinces have different economies, cultures, and resource allocations, leading to differences in green technological innovation efficiency. We should accelerate the rapid and balanced development of the economy, optimize the level of resource allocation, and at the same time, strengthen environmental protection and pollution control, formulate inclined policies for the central and western parts of the country, and improve the efficiency of the research and development stage and the transformation of scientific and technological achievements, to improve the efficiency of green technological innovation in all provinces and cities and all regions.

(2) China's high-tech industry should strengthen the management level of technological innovation, improve the utilization rate of resources, enhance the awareness of the integration of green technology into the R&D of enterprises, improve the conversion output of green innovation, actively promote the exchange of green technology, strengthen the integration of industry, academia, and research, and encourage the investment in the R&D of green technology, and at the same time, constantly adjust its technological direction, closely integrate green technology with social needs, and develop towards environment-friendly enterprises.

(3) The government should play an active role in enterprises' green innovation activities and set more flexible and applicable environmental regulation policies to guide enterprises' green technological innovation in all aspects of green inputs, energy consumption, environmental protection, and transformation of achievements, and at the same time, increase the regional synergistic development, pay more attention to the regions with low efficiency of green innovation, to tilt the resources to the inefficient areas, and incorporate the environmental regulation and pollution indexes into the evaluation index system of green technological innovation efficiency-technology innovation efficiency evaluation index system, to realize the typical leap of economic growth and environmental protection.

Acknowledgment

The authors wish to thank the 2022 Shenyang Philosophy and Social Science Planning Project, "Research on the Path and Countermeasures of Digital

Author Copy • Author Copy • Author Copy • Author Copy • Author Copy • Author Copy • Author Copy • Author Copy • Author Copy • Author Copy

Economy to Drive Shenyang's Industrial Structure Upgrading" (SY202237Y), for supporting this project.

Conflict of Interest

The authors declare no conflict of interest.

Notes on Contributors

Lei Liu, Master, Associate Professor, School of Management, Shenyang University of Technology. His research interests include science and technology innovation and management.

Li Zhang, Master candidate, School of Management, Shenyang University of Technology. His research interests include science and technology innovation and management.

Wei Xu, Doctor, Professor, School of Management, Shenyang University of Technology. His research interests include operations management and industrial engineering.

References

1. ZHANG W.Y., YU Q., YANG F.X., FAN H.Y. Evaluation of green innovation capacity of regional manufacturing industry under innovation-driven strategy - A complex network modeling analysis based on data from 30 regions. Industrial Technology and Economy, **37** (08), 86, **2018**.
2. YAO H.L., JIA R.K. Government Subsidies and Productivity of Resource Recycling Enterprises-An Empirical Study Based on Panel Threshold Effects of Listed Companies in China. Resource Science, **40** (11), 2280, **2018**.
3. CHEN S.Y. Construction and Measurement of Evaluation Indicator System for Green Innovation Capacity of High-Tech Industries. Statistics and Decision Making, **39** (03), 174, **2023**.
4. LI W., LIU Y.T., WU T.W. Impacts of environmental regulation and technological innovation on green economic efficiency of distribution industry in the context of green development. Research on Business Economy, **21**, 185, **2022**.
5. LUO X., ZHONG Y., GU X.Y.et al. A study on the impact of environmental regulation and the spatial and temporal leaps of heterogeneity in green technology innovation efficiency in China. Science and Technology Progress and Countermeasures, **39** (14), 52, **2022**.
6. BRAUN E., WIELD D. Regulation as a Means for the Social Control of Technology. Technology Analysis & Strategic Management, **6** (3), 259, **1994**.
7. GHISETTI C., RENNINGS K. Environmental innovations and profitability: how does it pay to be green? An empirical analysis of the German innovation survey. Journal of Cleaner Production, **75** (JUL.15), 106, **2014**.
8. HE X.G. Research on the optimal regulatory structure of green technological innovation--Based on the double interaction effect of R&D support and environmental regulation. Economic Management, **36** (11), 144, **2014**.
9. ZHANG F., REN S.J., YIN X.Q. Green technology innovation efficiency of high-tech industries and its scale quality threshold effect. Science and Technology Progress and Countermeasures, **37** (07), 59, **2020**.
10. LUO L.W., LIANG S.R. Green technology innovation efficiency and factor decomposition of regional industrial enterprises in China. China Population-Resources and Environment, **26** (09), 149, **2016**.
11. WANG Y., GONG X.S., LI J.J. Analysis of technological innovation efficiency and influencing factors of Xinjiang equipment manufacturing industry based on SFA model. Research on Science and Technology Management, **37** (12), 146, **2017**.
12. HE W. Evaluation of the comprehensive benefit of input and output in agricultural science and technology parks based on the DEA method. Statistics and Decision Making, **252** (24), 154, **2007**.
13. LU Y.H., SHEN C.C., TING C.T., WANG C.H. Research and development in productivity measurement: an empirical investigation of the high technology industry. Academic Journals, **4** (13), 2871, **2010**.
14. FRE R., GROSSKOPF S. Network DEA. Socio-Economic Planning Sciences, **3**, 249, **2000**.
15. LEWIS H.F., SEXTON T.R. Network DEA: Efficiency analysis of organizations with complex internal structure. Computers & Operations Research, **31** (9), 1365, **2004**.
16. ZHU H.H., YANG S.Q. Intellectual Property Protection, Technology R&D Investment and Two-stage Innovation Efficiency of Manufacturing Industry - A Comparative Analysis Based on Patent-intensive and Non-patent-intensive Manufacturing Industries. Modern Management Science, **333** (02), 50, **2022**.
17. TONE K. A slacks-based measure of efficiency in data envelopment analysis. European Journal of Operational Research, **130** (3), 498, **2001**.
18. LI H., LI W., YAO X.L. Research on spatial characteristics and dynamic convergence of total factor carbon emission efficiency in Chinese provinces. Science and Technology Management Research, **39** (19), 98, **2019**.
19. XU Y.Q., CHENG Y., WANG J.J. et al. Spatial and temporal evolution of carbon emission efficiency and influencing factors in Chinese low-carbon pilot cities. Journal of Natural Resources, **37** (05), 1261, **2022**.
20. YAN H.F., XIAO J., FENG B. Research on the spatial and temporal differentiation of industrial green technology innovation efficiency in the Yangtze River Economic Belt. Chongqing Social Science, **304** (03), 6, **2020**.
21. LAI Y.F., XIE P.J., YE L., MA X.R. Research on the Measurement and Influencing Factors of China's Regional Science and Technology Innovation Efficiency-Based on the Super-Efficient SBM-Malmquist-Tobit Model. Science and Technology Progress and Countermeasures, **38** (13), 37, **2021**.
22. CHEN W., ZHANG C.X., LI C.Y. et al. Research on the evaluation of technological innovation efficiency of high-tech industry based on the DEA-Malmquist index. Research on Science and Technology Management, **37** (23), 79, **2017**.
23. ZHANG L.K., ZHANG Y.P., LIANG Y. Measuring and evaluating the efficiency of green technology innovation of Chinese industrial enterprises – an analysis based on the SBM-Malmquist model of super-efficient network. Technological Economy, **41** (07), 13, **2022**.
24. ZHAO Q.Z., LIU J.X., CUI H.R. A study on the measurement and spatial convergence of technological

Author Copy • Author Copy • Author Copy • Author Copy • Author Copy • Author Copy • Author Copy • Author Copy • Author Copy • Author Copy • Author Copy • Author Copy • Author Copy

innovation efficiency in China's high-tech industries. Statistics and Decision Making, **39** (01), 183, **2023**.
25. JAFFE A.B., Palmer K.L. Environmental Regulation and Innovation: a Panel Data Study.The Review of Economics and Statistics, **79** (4), **1997**.
26. SUN Y.M., CHEN S.M. Spatio-temporal evolution pattern and driving factors of green technology innovation efficiency in the Yangtze River Delta region. Geography Research, **40** (10), 2743, **2021**.
27. LEY M., STUCKI T., WOERTER M. The Impact of Energy Prices on Green Innovation. Kof Working Papers, **37** (1), **2013**.
28. POPP D. International Innovation and Diffusion of Air Pollution Control Technologies: The Effects of NOX and SO2 Regulation in the US, Japan, and Germany. Journal of Environmental Economics & Management, **51** (1), 46, **2006**.
29. LI D.D. A study on the impact of government R&D subsidies on firms' innovation performance – Based on the perspectives of firm size and property rights heterogeneity. Economic Journal, **9** (01), 141, **2022**.
30. ZHANG L., HUANG L.Q. Measurement of green technology innovation efficiency of Chinese industrial enterprises and its temporal and spatial differentiation characteristics – an analysis based on the improved three-stage SBM-DEA model. Statistics and Information Forum,**35** (12), 50, **2020**.
31. SUN Y.N., FEI J.H. Measurement, sources of differences and causes of green productivity of heavily polluting enterprises. China Population-Resources and Environment, **31** (11), 102, **2021**.
32. ALLEN B., ZHENG Y.L., Liu A.A. Efficacy of Command-and-Control and Market-Based Environmental Regulation in Developing Countries. Annual Review of Resource Economics, **10** (1), annurev-resource-100517-023144-, **2018**.
33. OH D.H. A global Malmquis-Luenberge productivity index. Journal of Productivity Analysis, **34** (3), 183, **2010**.
34. Y. H., CHUANG. Productivity and Undesirable Outputs: A Directional Distance Function Approach. Journal of Environmental Management, **51** (3), 229, **1997**.
35. XIAO R.Q., SHEN L., QIAN L. Research on green innovation efficiency of industrial enterprises and its influencing factors in provinces along the "Belt and Road". Soft Science, 3**4** (08), 37, **2020**.
36. LI B., PENG X. Research on the spatial heterogeneous effect of environmental regulatory tools – a spatial econometric analysis based on the perspective of government function transformation. Industrial Economics Research, **67** (06), 38, **2013**.
37. MILANI S. The Impact of Environmental Policy Stringency on Industrial R&D Conditional on Pollution Intensity and Relocation Costs. Environmental and Resource Economics, **68** (3), 595, **2017**.
38. SHIMSHARK J.P., WARD M.B. Regulator reputation, enforcement, and environmental compliance. Journal of Environmental Economics & Management, **50** (3), 519, **2005**.
39. TESTA F., IRALDO F., FREY M. The effect of environmental regulation on firms' competitive performance: the case of the building & construction sector in some EU regions. Journal of Environmental Management, **92** (9), 2136, **2011**.
40. YUAN G.P., LI J.J. Impact and spillover effects of mixed ownership reform on green innovation in state-owned enterprises. China Population-Resources and Environment, **33** (07), 180, **2023**.
41. WU Z H., LI T. Research on the spatial distribution of comprehensive performance of high-tech development zones – an analysis based on the natural breakpoint method. Statistics and Information Forum, **28** (3), 82, **2013**.
42. FANG S.M., HUANG Y. Spatio-temporal evolution and coupled coordination of tourism efficiency and scale in the Yangtze River Economic Belt. Journal of Geography, **75** (08), 1757, **2020**.
43. LUO Z.H., TAO J. Evaluation of green technology innovation efficiency and analysis of influencing factors in high-tech industries - based on SBM-Tobit model. Journal of Heilongjiang Institute of Technology (General Edition), **23** (04), 116124, **2023**.
44. ZHANG T., WANG W.R., YAO Y.C. Research on the Evaluation of Science and Technology Innovation Efficiency of 31 Provinces (autonomous regions and municipalities) and Key Provinces and Regions in China Based on the Super-Efficient SBM-Malmquist Model. Science and Technology Management Research, **43** (03), 87, **2023**.
45. ZHANG F., SHI Z.W., SONG X.N., YAN X.X. Green technology innovation efficiency of advanced manufacturing industry and its environmental regulation threshold effect. Science and Technology Progress and Countermeasures, **36** (12), 62, **2019**.
