# Supplementary material for: Research on the enhancement path of green technology innovation efficiency under the group perspective
Source: PLoS One. 2024 Aug 15;19(8):e0306936. doi: 10.1371/journal.pone.0306936 (PMC11326581; doi:10.1371/journal.pone.0306936)
Supplement: Attachment — Submitted filename: Response to reviewers.doc [file pone.0306936.s003.doc]

Dear reviewers

Thank you for reviewing our manuscript, we greatly appreciate your constructive suggestions and have revised the manuscript accordingly. We have now clarified the applicability of the manuscript in more detail and have carefully revised the relevant wording issues you raised. And, we have carefully examined the manuscript and carefully revised similar issues that have arisen elsewhere. Thank you again for your valuable suggestions.

| Reviewers' recommendations | Author's Response. |
| --- | --- |
| Pag. 1, line 11-19: the paragraph is too long and syntactically complex. Make shorter sentences.  original sentence:“Green transformation and upgrading is the road from high-speed development to high-quality, sustainable development; the party's 18th National Congress emphasized the need to take green development as an essential way to transform the economy and promote the development of high-quality society and to achieve green transformation, technology is a solid supporting condition, technological innovation in the innovation process is constantly integrated into the green concept, so that the green technological innovation has become the first driving force to lead the green development, not only can it bring technological progress can also protect the ecological environment” | Thank you very much for your suggestion, the sentence is complex and lengthy, I have described the sentence briefly and highlighted it in yellow in the article, here is the revised sentence.  revised sentence：“Green Transformation and Upgrading is the Way from High-Speed Development to High Quality and Sustained Development, technological innovation in the innovation process is constantly integrated into the green concept to bring technological progress and protect the ecological environment” |
| Pag. 2, line 38-39: the sentence is not clear “three aspects of society, economy, and environment.” And the third? Rephrase the sentence or explain better.  original sentence：“Green technological innovation is different from ordinary innovation, not only to use innovation to produce knowledge-based innovations and create as many economic benefits as possible, but also to reduce pollution emissions and realize energy conservation and environmental protection through innovation, and more importantly, to rely on innovation to learn the coordinated symbiosis of the three aspects of society, economy, and environment.” | Thank you very much for your advice, I apologise for my carelessness and have now amended the sentence as you suggested,and highlighted the sentence in yellow in the text  revised sentence:“Green technological innovation is different from ordinary innovation. Innovation should generate knowledge and maximum economic benefits, reduce pollution emissions, save energy, and protect the environment, and more important is to rely on innovation to achieve a harmonious symbiosis between society, the economy and the environment” |
| The work is specifically oriented towards experience and reality related to Chines perspective; this may encounter occasional difficulties in replicating the conclusion of the work in different realities. | Thank you for your suggestion, and we have added the word "China" to the abstract to make it clear that the article is based on Chinese research and to emphasise its applicability. You can see the corresponding changes in the Abstract section of the manuscript. |

Thank you very much for your attention and time.

Your sincerely

Li Zhang

Shenyang University of Technology
