# Supplementary material for: Research on the enhancement path of green technology innovation efficiency under the group perspective
Source: PLoS One. 2024 Aug 15;19(8):e0306936. doi: 10.1371/journal.pone.0306936 (PMC11326581; doi:10.1371/journal.pone.0306936)
Supplement: Attachment — Submitted filename: Comments.docx [file pone.0306936.s004.docx]

This study is innovative but still needs further revision.

1. The authors are expected to add references to support the literature review section and not write from their own knowledge and experience.

2. The authors should have expressed more clearly the theoretical and practical implications of this study.

3. The authors should have added more compelling information in the data sources section.

4. The writer should revise the language of the paper as a whole; there are many grammatical errors in this paper. It is recommended that the writer seek the help of a native English speaker.
