# Supplementary material for: Research on the enhancement path of green technology innovation efficiency under the group perspective
Source: PLoS One. 2024 Aug 15;19(8):e0306936. doi: 10.1371/journal.pone.0306936 (PMC11326581; doi:10.1371/journal.pone.0306936)
Supplement: Attachment — Submitted filename: 23-35015 comments.docx [file pone.0306936.s005.docx]

1. In terms of theoretical framework construction, the paper is slightly thin in theoretical elaboration and model construction. It is suggested that the authors strengthen the sorting and elaboration of related theories to further clarify the connotation and influencing factors of green technology innovation efficiency, so as to better support the subsequent empirical analysis and conclusions.

2. The source and selection of data is critical to the accuracy and reliability of the study results. Authors need to clearly state the reliability of the data sources and how the data were processed to ensure the rigor of the study.

3. In terms of research methodology, the thesis adopts a fuzzy set qualitative comparative analysis method (fs-QCA) based on group theory to explore the complex causal mechanisms and grouping paths that affect the efficiency of green technological innovation among enterprises, governments and markets. The choice of this method is innovative but may have some limitations in practical application. The authors need to analyze the applicability of the method in depth and compare it with other possible methods to verify the robustness of the findings.

4. It is recommended to increase the limitations of the paper.
