# Supplementary material for: Research on the enhancement path of green technology innovation efficiency under the group perspective
Source: PLoS One. 2024 Aug 15;19(8):e0306936. doi: 10.1371/journal.pone.0306936 (PMC11326581; doi:10.1371/journal.pone.0306936)
Supplement: Attachment — Submitted filename: Response to reviewers.doc [file pone.0306936.s006.doc]

Dear reviewers

Thank you for reviewing our manuscript. We appreciate your constructive suggestions and have revised it accordingly. We have now clarified the applicability of the manuscript in more detail and have carefully revised the relevant issues you raised. In addition, we have scrutinized the manuscript and made careful revisions to similar problems that have arisen elsewhere. Thank you again for your valuable suggestions. Below are the answers to each of your valuable comments, and you can see the revised manuscript or manuscript for specific changes.

Q1: In terms of theoretical framework construction, the paper is slightly thin in theoretical elaboration and model construction. It is suggested that the authors strengthen the sorting and elaboration of related theories to further clarify the connotation and influencing factors of green technology innovation efficiency, so as to better support the subsequent empirical analysis and conclusions.

Response: Thank you very much for your suggestions. With your suggestions, we have carefully sorted out the relevant theories to make them more logical; we have also used more ink to construct the model and carefully elaborated on the profound influence of enterprises, governments, and markets on the efficiency of green technological innovation. We hope that this modification can better support the empirical content in the later part of the paper.

Q2: The source and selection of data is critical to the accuracy and reliability of the study results. Authors need to clearly state the reliability of the data sources and how the data were processed to ensure the rigor of the study.

Response: Thank you very much for your suggestion, yes, the source of data is very important, our data in this thesis mainly comes from CHINA STATISTICAL YEARBOOK ON science and technology and CHINA STATISTICAL YEARBOOK ON environment , the former is the Chinese The former is a statistical information book reflecting the situation of science and technology activities in China, edited by the Department of Social, Scientific and Cultural Industry Statistics of the National Bureau of Statistics of China and the Department of Strategic Planning of the Ministry of Science and Technology, which includes the annual statistics on science and technology of 31 provinces, autonomous regions, municipalities directly under the central government, and the relevant departments of the State Council of China, while the latter is an annual comprehensive statistical information book reflecting the basic situation of China's environment, edited by the National Bureau of Statistics of China, the Ministry of Ecological and Environmental Protection, and other relevant ministries. The latter is an annual comprehensive statistical information reflecting the basic situation of various fields of the environment in China, edited by the National Bureau of Statistics of China, the Ministry of Ecology and Environment, and other relevant ministries. The source of data is reliable. Due to the large amount of data involved, the processing of the data is placed in Section 5.1.2, Selection of Indicators, and 5.2, Conditional Variable Measurement of the article.

Q3: In terms of research methodology, the thesis adopts a fuzzy set qualitative comparative analysis method (fs-QCA) based on group theory to explore the complex causal mechanisms and grouping paths that affect the efficiency of green technological innovation among enterprises, governments and markets. The choice of this method is innovative but may have some limitations in practical application. The authors need to analyze the applicability of the method in depth and compare it with other possible methods to verify the robustness of the findings.

Response: Thanks to your suggestion, we have innovatively chosen the QCA method to explore the complex causal mechanisms of green technological innovation, and this method has been selected after much deliberation. In section 4.1 of the thesis, we have analyzed the applicability of this method in depth. At your suggestion, we have conducted three stability tests in section 6, Robustness Tests, using the two main methods: the Adjustment Consistency thresholds, adjusting calibration anchors, and grouping analyses across time periods to make our findings more stable.

Q4: It is recommended to increase the limitations of the paper.

Response: Thank you very much for your suggestion. We recognized the article's limitations at your suggestion, and in section 7, we have added a description.

Dear reviewer, thank you again for your valuable suggestions, which made our article more full and readable, and the quality of the article has been taken to the next level. Thank you very much, and have a nice day!

Thank you very much for your attention and time.

Your sincerely

Li Zhang

Shenyang University of Technology
